# Performance of Reasoning and Comparator Large Language Models on Nephrology Multiple-choice Questions

**DOI:** 10.31662/jmaj.2025-0603

**Published:** 2026-08-14

**Authors:** Fumiya Kitano, Mamoru Masaki, Daisuke Ichikawa, Yugo Shibagaki, Ryunosuke Noda

**Affiliations:** 1 Division of Nephrology and Hypertension, Department of Internal Medicine, St. Marianna University School of Medicine, Kawasaki, Japan

**Keywords:** generative artificial intelligence, large language models, multiple-choice questions, nephrology, reasoning models

## Abstract

**Introduction:**

Performance of large language models in medicine is improving; yet, it remains unclear how the advantage of reasoning models depends on task characteristics in nephrology.

**Methods:**

We evaluated four large language models in two families—OpenAI (GPT-5 reasoning and GPT-4o comparator) and Google (Gemini 2.5 Pro reasoning and Gemini 2.0 Flash comparator)—on 209 self-assessment questions for nephrology board renewal published by the Japanese Society of Nephrology. Questions were categorized by question type (general vs. clinical), taxonomy (recall, interpretation, and problem-solving), and image inclusion (non-image vs. image). Models were assessed via application programming interface; no generation parameters were configured; only the model was selected. Images were provided as PNG files. Accuracy used Wilson 95% confidence intervals (CIs); paired comparisons used McNemar’s exact test. Primary analyses used logistic generalized linear mixed models with fixed effects, random intercepts, and prespecified interactions.

**Results:**

Overall accuracy was 87.6% (183/209, 95% CI 82.4-91.4) for GPT-5 and 83.7% (175/209, 95% CI 78.1-88.1) for Gemini 2.5 Pro versus 69.9% (146/209, 95% CI 63.3-75.7) for GPT-4o and 62.7% (131/209, 95% CI 55.9-69.0) for Gemini 2.0 Flash. Paired analyses favored the reasoning models, with odds ratios of 6.29 for OpenAI and 7.29 for Google (both p <0.001). Adjusted odds ratios for reasoning versus comparator models at the reference strata (general, recall, and non-image questions) were 5.00 for OpenAI and 7.28 for Google (both p <0.001). Interactions showed stronger effects in clinical questions for OpenAI and taxonomy-dependent effects for Google; no significant modification by image inclusion was observed.

**Conclusions:**

The reasoning models tested here outperformed their comparator models on nephrology board–style multiple-choice questions, with advantages that varied by question type. These findings support further evaluation of reasoning models as supplementary tools for nephrology board–recertification preparation, while underscoring that direct clinical use requires task-specific validation.

## Introduction

Large language models (LLMs) have brought groundbreaking advances in natural language processing, demonstrating high performance in diverse tasks such as summarization, translation, question answering, and complex text generation ^(1), (2)^. In the medical field, they are expected to contribute widely to applications including medical education support, clinical decision-making, and research efficiency ^(3), (4), (5)^. In particular, OpenAI’s GPT series and Google’s Gemini series have shown promising results in the United States Medical Licensing Examination, the Japanese National Medical Licensing Examination, and various specialty board examinations, demonstrating both medical knowledge and problem-solving ability ^(6), (7), (8), (9), (10), (11), (12), (13), (14)^.

Recently, newer LLMs referred to as reasoning models have been introduced, with the stated aim of improving performance on tasks requiring multistep reasoning ^(15), (16), (17), (18), (19)^. The broader literature on LLM reasoning has described approaches such as chain-of-thought prompting, self-consistency, and optimization for generating stepwise reasoning processes; however, the proprietary implementations of the models evaluated in this study were not examined. Some reasoning models have been reported to provide intermediate explanatory outputs, which may increase transparency and facilitate error analysis, whereas they also present risks, such as increased computational costs and the tendency to produce plausible but incorrect reasoning (hallucinations) ^(20)^. In this study, we use the term “reasoning models” operationally to refer to newer models selected for comparison within each vendor family because of their reasoning-related features, and this terminology is intended for descriptive comparison and does not imply causal attribution.

Prior studies have reported that comparator models such as GPT-4 may demonstrate certain capabilities in nephrology board–level questions, suggesting that LLMs could serve as useful tools for education and clinical practice support ^(13), (14)^. Nevertheless, nephrology is a highly specialized field that involves complex physiology related to systemic homeostasis, a broad disease spectrum, and the management of multiple comorbidities. In this domain, advanced clinical reasoning is indispensable, including the interpretation of test results, differential diagnosis based on pathophysiology, and formulation of individualized treatment strategies, all of which require complex reasoning and therefore provide a relevant context for evaluating the performance of different model types. However, it has not been sufficiently investigated how the superiority of reasoning models is influenced by task characteristics in nephrology. Therefore, we aimed to quantify the performance gap between reasoning and comparator models in nephrology and to analyze how this gap is modified by specific question characteristics.

## Materials and Methods

### Self-Assessment Questions for Nephrology Board Renewal (SAQ-NBR)

The SAQ-NBR question set consists of nephrology-related multiple-choice questions (MCQs) written in Japanese, administered annually by the Japanese Society of Nephrology ^(21), (22)^. The SAQ-NBR is used as one component of the nephrology board renewal credit system and is designed to comprehensively assess knowledge across the full spectrum of kidney diseases. An accuracy rate of ≥60% is required for awarding renewal credits ^(21)^. We analyzed 209 of 210 MCQs drawn from 10 annual SAQ-NBR examinations administered between 2014 and 2023, after excluding one item that had been officially invalidated by the Japanese Society of Nephrology. Each question was annotated along four prespecified categories to capture task characteristics, defined in line with prior literature ^(13), (14)^. Annotations were performed by two authors (Fumiya Kitano and Ryunosuke Noda) using prespecified definitions based on our prior framework and a previously described taxonomy ^(23)^. The annotators independently reviewed question stems, answer choices, and associated images, and discrepancies were resolved by consensus. Annotations were based solely on question content and conducted independently of model outputs. Interrater agreement was not formally assessed, as annotations were finalized through consensus. Question type was classified as general (testing factual knowledge) or clinical (testing application in patient scenarios), and taxonomy (testing increasing levels of cognitive demand) was divided into recall, interpretation, and problem-solving (following the Japanese National Medical Examination taxonomy framework created by the Japan Medical Association ^(23)^); image inclusion was defined as non-image– versus image-based questions (pathology micrographs or radiological images, as well as schematic diagrams), and subspecialty was mapped to eight domains (chronic kidney disease/end-stage kidney disease, acute kidney injury, glomerular diseases, tubulointerstitial diseases, hypertension/vascular diseases, water/electrolyte/acid-base disorders, autosomal dominant polycystic kidney disease/urology, and basic medicine) using the Japanese Society of Nephrology case experience list ^(24)^.

### LLMs and evaluation settings

Two LLM families were evaluated, each comprising a reasoning model and a comparator model: OpenAI (GPT-5 [gpt-5-2025-08-07] as the reasoning model and GPT-4o [gpt-4o-2024-11-20] as the comparator) and Google (Gemini 2.5 Pro [gemini-2.5-pro-preview-03-25] as the reasoning model and Gemini 2.0 Flash [gemini-2.0-flash-001] as the comparator). To approximate production-like use, all models were accessed via application programming interface (API), and in the API calls we specified only the exact model identifier without explicitly setting generation parameters such as temperature or top-p. Vendor-documented default parameters applied when such settings were omitted are summarized in Supplementary Table S1. GPT-4o, Gemini 2.5 Pro, and Gemini 2.0 Flash were evaluated in April 2025, whereas GPT-5 was evaluated in August 2025. All questions were presented in Japanese, preceded by a standardized prompt: “We will now present a nephrology-related question. Please provide your answer and explanation.” For image questions, the source PDF file was displayed at its original magnification, and images were captured using the standard Windows Snipping Tool and saved as PNG files, without additional preprocessing. The PNG images were then input together with the question text as a multimodal API request. For each model–question pair, the outcome was defined as a binary variable (correct = 1, incorrect = 0). Correctness was adjudicated against the official answer keys issued by the Japanese Society of Nephrology.

### Statistical analysis

We first computed overall and category-wise accuracy with Wilson 95% confidence intervals (CIs). Within each family, paired comparisons between the reasoning and comparator models were performed using McNemar’s exact test, and odds ratios (ORs) based on discordant pairs were reported. The primary analyses used logistic generalized linear mixed models (GLMMs) fitted separately by family. Fixed effects included model (reasoning vs. comparator), question type (general vs. clinical), taxonomy (recall, interpretation, and problem-solving), and image inclusion (image vs. non-image). To assess effect modification, we prespecified interaction terms between model and question type, model and taxonomy, and model and image, with inference based on Wald tests. To accommodate the nested data structure, we included random intercepts for question and for case scenario in chained or related items. Because of concerns about multiplicity and estimator stability, subspecialty was not entered as an interaction term in the GLMMs. We reported adjusted ORs (aORs) with 95% CIs as the principal effect measures. For visualization, simple-effect aORs were derived from the GLMM coefficients and displayed in forest plots for the prespecified stratifications. All analyses were conducted in Python (version 3.11.13; Python Software Foundation), and a two-sided p <0.05 was considered statistically significant.

For the qualitative error analysis, we identified questions answered incorrectly by all four models and conducted a descriptive review of their content and potential sources of failure. This review was performed by two authors (Fumiya Kitano and Ryunosuke Noda), who examined the items and discussed their interpretations to reach a consensus summary. Because this analysis was exploratory and interpretive in nature, we did not apply a formal coding framework or calculate interrater agreement. This analysis was intended to generate hypotheses regarding common failure modes rather than to provide a systematic classification.

## Results

### Overall performance and model comparisons

For the 209 questions analyzed, the overall accuracy of the reasoning models was 87.6% for GPT-5 (183/209, 95% CI 82.4-91.4) and 83.7% for Gemini 2.5 Pro (175/209, 95% CI 78.1-88.1). For the comparator models, GPT-4o achieved 69.9% (146/209, 95% CI 63.3-75.7) and Gemini 2.0 Flash 62.7% (131/209, 95% CI 55.9-69.0). Thus, in both the OpenAI and Google families, the reasoning models outperformed the comparator models (Figure 1). In paired comparisons on identical questions, GPT-5 was exclusively correct on 44 questions, while GPT-4o was exclusively correct on seven (OR 6.29, 95% CI 3.25-16.00; p <0.001). Similarly, Gemini 2.5 Pro was exclusively correct on 51 questions compared with seven for Gemini 2.0 Flash (OR 7.29, 95% CI 3.83-18.33; p <0.001). In both families, the reasoning models were significantly superior (Table 1).

**Figure 1.**
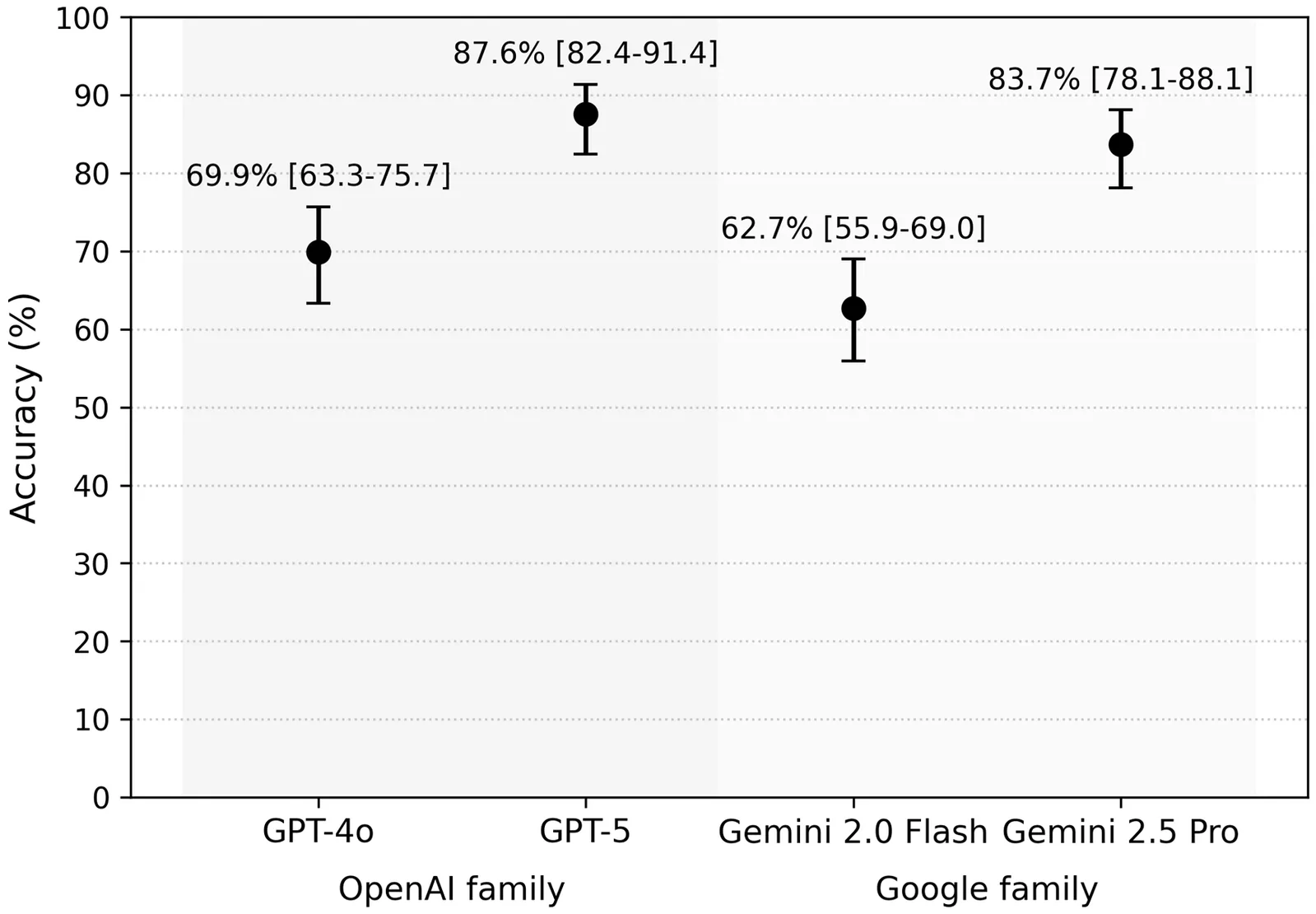
Overall accuracy on Self-Assessment Questions for Nephrology Board Renewal. For each model, the dot shows the percentage of correct answers, and the error bars show the 95% Wilson confidence intervals. In both model families, the reasoning models (GPT-5 and Gemini 2.5 Pro) outperformed their respective comparator models (GPT-4o and Gemini 2.0 Flash).

**Table 1.** Paired Comparison of Performance between Reasoning and Comparator Models.

Comparisons	Reasoning model: only correct (n)	Comparator model: only correct (n)	Risk difference	Odds ratio [95% CI]	p-Value
OpenAI (GPT-5 vs. GPT-4o)	44	7	0.18	6.29 [3.25-16.00]	<0.001
Google (Gemini 2.5 Pro vs. 2.0 Flash)	51	7	0.21	7.29 [3.83-18.33]	<0.001

Notes: This paired analysis compares the performance of two model types on the same set of questions. The column “Reasoning model: only correct (n)” indicates the number of questions answered correctly by the reasoning model but incorrectly by the comparator model. Conversely, “Comparator model: only correct (n)” indicates the number of items answered correctly by the comparator model but incorrectly by the reasoning model. The odds ratio is calculated as the ratio of these two discordant pair counts. p-Values were derived from McNemar’s exact test. CI: confidence interval.

### Accuracy by question categories

When stratified by question categories, the reasoning models consistently achieved higher accuracy across all major categories (question type, taxonomy, and image inclusion) compared with the comparator models. In the OpenAI family, the performance gap was particularly notable in clinical questions, where GPT-5 achieved 86.7% (95% CI 78.6-92.1) versus 65.3% (95% CI 55.5-74.0) for GPT-4o. In the Google family, a large gap was observed in recall questions, with Gemini 2.5 Pro at 86.1% (95% CI 78.3-91.4) compared with 62.0% (95% CI 52.6-70.6) for Gemini 2.0 Flash. Detailed stratified accuracies, including by subspecialty, are provided in Table 2.

**Table 2.** The Proportion of the Correct Answers of GPT-4o, GPT-5, Gemini 2.0 Flash, and Gemini 2.5 Pro by four categories on the Self-Assessment Questions for Nephrology Board Renewal.

Categories	GPT-4o	GPT-5	Gemini 2.0 Flash	Gemini 2.5 Pro
*Question type*				
General	73.9% [65.0-81.1] (82/111)	88.3% [81.0-93.0] (98/111)	64.0% [54.7-72.3] (71/111)	85.6% [77.9-90.9] (95/111)
Clinical	65.3% [55.5-74.0] (64/98)	86.7% [78.6-92.1] (85/98)	61.2% [51.3-70.3] (60/98)	81.6% [72.8-88.1] (80/98)
*Taxonomy*				
Recall	73.1% [64.1-80.6] (79/108)	88.9% [81.6-93.5] (96/108)	62.0% [52.6-70.6] (67/108)	86.1% [78.3-91.4] (93/108)
Interpretation	66.1% [53.0-77.1] (37/56)	82.1% [70.2-90.0] (46/56)	60.7% [47.6-72.4] (34/56)	76.8% [64.2-85.9] (43/56)
Problem-solving	66.7% [52.1-78.6] (30/45)	91.1% [79.3-96.5] (41/45)	66.7% [52.1-78.6] (30/45)	86.7% [73.8-93.7] (39/45)
*Image inclusion*				
Non-image	71.6% [64.4-77.9] (121/169)	89.3% [83.8-93.2] (151/169)	61.5% [54.0-68.5] (104/169)	83.4% [77.1-88.3] (141/169)
Image	62.5% [47.0-75.8] (25/40)	80.0% [65.2-89.5] (32/40)	67.5% [52.0-79.9] (27/40)	85.0% [70.9-92.9] (34/40)
*Subspecialty*				
CKD/ESKD	71.1% [56.6-82.3] (32/45)	84.4% [71.2-92.3] (38/45)	51.1% [37.0-65.0] (23/45)	80.0% [66.2-89.1] (36/45)
AKI	70.0% [39.7-89.2] (7/10)	90.0% [59.6-98.2] (9/10)	70.0% [39.7-89.2] (7/10)	80.0% [49.0-94.3] (8/10)
Glomerular diseases	64.9% [51.9-76.0] (37/57)	86.0% [74.7-92.7] (49/57)	71.9% [59.2-81.9] (41/57)	89.5% [78.9-95.1] (51/57)
Tubulointerstitial diseases	63.2% [41.0-80.9] (12/19)	94.7% [75.4-99.1] (18/19)	47.4% [27.3-68.3] (9/19)	89.5% [68.6-97.1] (17/19)
Hypertension/vascular diseases	66.7% [43.7-83.7] (12/18)	72.2% [49.1-87.5] (13/18)	66.7% [43.7-83.7] (12/18)	72.2% [49.1-87.5] (13/18)
Water/electrolyte/acid-base disorders	60.9% [40.8-77.8] (14/23)	95.7% [79.0-99.2] (22/23)	65.2% [44.9-81.2] (15/23)	82.6% [62.9-93.0] (19/23)
ADPKD/urology	91.7% [64.6-98.5] (11/12)	83.3% [55.2-95.3] (10/12)	75.0% [46.8-91.1] (9/12)	75.0% [46.8-91.1] (9/12)
Basic science	84.0% [65.3-93.6] (21/25)	96.0% [80.5-99.3] (24/25)	60.0% [40.7-76.6] (15/25)	88.0% [70.0-95.8] (22/25)

Notes: Stratified accuracy by category (Wilson 95% CI). Values are presented as percentage [95% CI] (correct/total). ADPKD: autosomal dominant polycystic kidney disease; AKI: acute kidney injury; CI: confidence interval; CKD: chronic kidney disease; ESKD: end-stage kidney disease.

### Multivariable analysis adjusted for question categories

To assess whether the superiority of the reasoning models was modified by question characteristics, we fitted multivariable logistic GLMMs with prespecified interaction terms (Table 3). In the OpenAI family, we found a significant interaction between the model and question type (p for interaction = 0.007). The advantage of the reasoning model was significantly greater for clinical questions (aOR 13.94, 95% CI 5.68-34.25) compared with general questions (aOR 5.00, 95% CI 3.00-8.35) (Figure 2a). Conversely, in the Google family, a significant interaction was observed between the model and taxonomy (p for interaction = 0.026). The reasoning model’s advantage was significantly smaller for interpretation questions (aOR 2.56, 95% CI 1.02-6.45) compared with the reference category of recall questions (aOR 7.28, 95% CI 4.60-11.52) (Figure 2b). Finally, no significant interaction was found with image inclusion in either the OpenAI family (p for interaction = 0.219) or the Google family (p for interaction = 0.786), indicating that the reasoning models did not provide a statistically significant additional benefit for questions containing medical images compared with text-only questions.

**Table 3.** Comparison of Large Language Model Performance by Multivariable Generalized Linear Mixed Model.

Parameters	OpenAI (GPT-5 vs. GPT-4o)	Google (Gemini 2.5 Pro vs. 2.0 Flash)
aOR [95% CI]	5.00 [3.00-8.35]	7.28 [4.60-11.52]
p-Value (main effect)	<0.001	<0.001
*p for interaction*		
Question type	0.007	0.118
Taxonomy	0.334	0.026
Image inclusion	0.219	0.786

Notes: The aOR represents the odds of a correct answer for the reasoning model compared to the comparator model (reference). The aORs for the model effect represent the effect at the reference strata of each interaction term (general questions, recall taxonomy, and non-image questions). The model was adjusted for question characteristics (question type, taxonomy, and image inclusion). The p for interaction evaluates whether the effect of the model differs across the subgroups of each question characteristic. Random effects for Item (question ID) and ContextID (case ID) were included to account for data clustering. aOR: adjusted odds ratio; CI: confidence interval.

**Figure 2.**
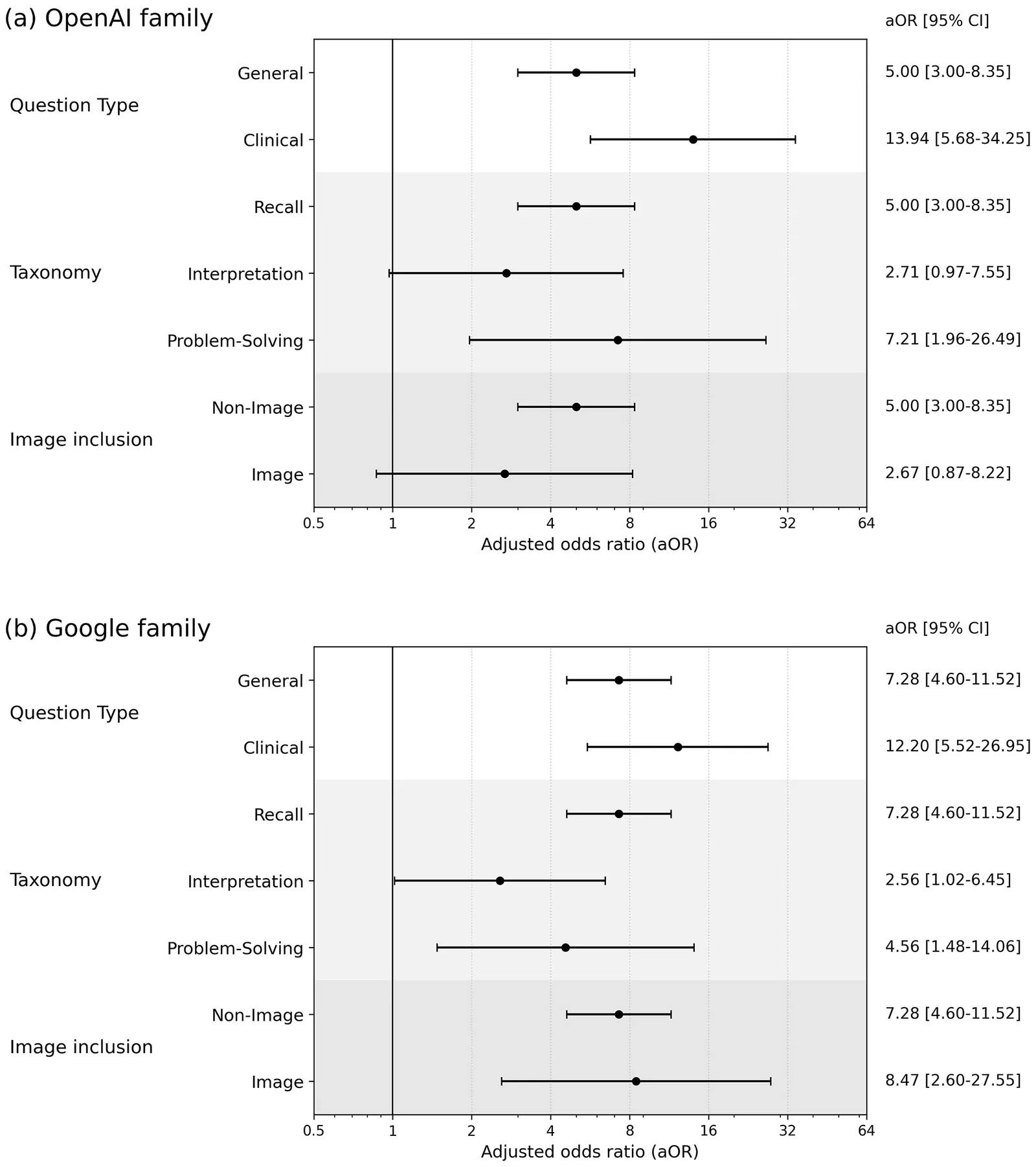
Subgroup effects of reasoning models versus comparator models. Forest plots display aORs for a correct answer by the reasoning models compared with the comparator models within (a) the OpenAI family and (b) the Google family. Estimates come from a logistic generalized linear mixed model adjusting for question characteristics (question type, taxonomy, and image inclusion), with random intercepts for each question and case scenario. Subgroup values are simple effects relative to the reference categories “general,” “recall,” and “non-image.” An aOR >1 favors the reasoning model. Error bars indicate 95% confidence intervals, and the x-axis is on a log scale. aOR: adjusted odds ratio.

### Qualitative analysis of incorrect response patterns

Of the 209 questions, 105 (50.2%) were answered correctly by all four models. Twenty-five questions (12.0%) were answered correctly by both reasoning models but incorrectly by both comparator models. In contrast, there were no questions (0%) where both comparator models answered correctly and both reasoning models failed. Fifteen questions (7.2%) were answered incorrectly by all four models. A qualitative review of these 15 questions revealed that they frequently involved knowledge specific to Japanese clinical guidelines or domestic epidemiological data (Table 4).

**Table 4.** Analysis of Representative Questions Incorrectly Answered by All Models.

Question summaries	Error category	Error of models
Risk stratification in the Japanese Society of Hypertension Guidelines for the Management of Hypertension 2019	Japan-specific guidelines	The models identified “obesity” as a major risk factor based on international standards, failing to reflect the unique risk stratification of the Japanese Society of Hypertension 2019 guidelines where obesity is not included in the primary/secondary risk tiers.
Epidemiology of anemia in patients with non-dialysis CKD in Japan	Japan-specific epidemiological data	The models judged based on general clinical trends (anemia worsens as CKD progresses), failing to reflect Japan-specific epidemiological data from a large-scale JSDT survey showing that approximately half of patients with G4/G5 CKD achieve the target hemoglobin level.
Malignancies in dialysis patients in Japan	Japan-specific epidemiological data	The models responded based on general cancer epidemiology, failing to reflect the unique epidemiology from Japanese registry data, which show that genitourinary malignancies are the most common cancer in male dialysis patients.
Population attributable risk of cardiovascular mortality in the EPOCH-JAPAN study	Japan-specific epidemiological data	While acknowledging the importance of blood pressure control, the models failed to provide the specific population attributable risk (~40%) reported by the Japanese EPOCH-JAPAN study.

Notes: This is a qualitative analysis of questions that all four language models answered incorrectly. For each representative problem, the table provides a summary of the topic, classifies the error category, and describes the presumed reasoning for the models’ failure. The errors frequently stemmed from a reliance on international or generalized medical knowledge, which was inconsistent with clinical guidelines or epidemiological data specific to Japan. CKD: chronic kidney disease; EPOCH-JAPAN: Evidence for Cardiovascular Prevention from Observational Cohorts in Japan; JSDT: Japanese Society for Dialysis Therapy.

## Discussion

In this study using Japanese nephrology board renewal questions, reasoning models (GPT-5 and Gemini 2.5 Pro) consistently outperformed comparator models (GPT-4o and Gemini 2.0 Flash) in both the OpenAI and Google families. Their advantage was not consistent across all question types: in multivariable analyses, the magnitude of this advantage varied by question characteristics, with the OpenAI family showing particularly strong gains on clinical questions and the Google family showing notable improvements on recall questions. Questions testing Japan-specific guidelines or epidemiology were frequently answered incorrectly by all models, underscoring important challenges that must be addressed before these systems can be reliably applied in real-world clinical practice.

The overall superiority of reasoning models in this study is consistent with prior evaluations in which reasoning models achieved higher answer accuracy. For example, o1 pro achieved 81.3% answer accuracy compared with 51.2% for GPT-4 on nephrology board renewal MCQs ^(14)^, and o3 achieved 83.3% accuracy compared with 70.0% for ChatGPT-3.5 on an original set of pharmacotherapy MCQs ^(25)^. In our study, the advantage of reasoning models was greater for clinical questions than for general questions, especially within the OpenAI family. This pattern suggests that these models may be particularly effective in tasks that require integrating multiple pieces of patient information and test data within a clinical context to approximate clinical decision-making. A similar possibility has been highlighted in an editorial discussing o1-preview, which suggests that its enhanced chain-of-thought reasoning could be beneficial for complex clinical case–based diagnostic and management tasks compared with GPT-4 ^(26)^.

Despite these overall performance gains, the benefits of reasoning models were not uniform across all task types, highlighting important limitations of current systems. In the Google family, the relative advantage of the reasoning model was greater for recall questions and smaller for interpretation questions. In contrast, the OpenAI family showed no statistically significant taxonomy interaction. Reasoning models can exhibit hallucinations, which can be accompanied by breakdowns in the reasoning process ^(20)^. This may partly contribute to the smaller relative advantage observed for interpretation questions in the Google family. Prior work has also shown that LLM performance may decline on more complex, context-dependent reasoning tasks ^(14)^. One diagnostic accuracy study found that models such as GPT-4 achieved substantially lower scores on reasoning-type questions than on knowledge-based questions ^(27)^, and a separate investigation of clinical decision-making showed that nonreasoning models displayed notable limitations in multistep, real-world–like scenarios even when they achieved high scores on medical licensing–style examinations ^(28)^.

Our analysis revealed no significant interaction between reasoning model and image inclusion, even though image questions were expected to rely more on higher-level visual–textual reasoning. One possible explanation is that the integration of visual information may still be suboptimal. Even in the latest reasoning models such as GPT-5 and o3, recent studies have reported that performance in image-based spot-diagnosis tasks remains far below that of human specialists ^(29)^. Similarly, evaluations using the New England Journal of Medicine Image Challenge and various specialty board examinations have shown that adding image inputs does not necessarily improve performance and in some instances may offer no advantage over text-only inputs ^(30)^. Other studies have further suggested that text-only descriptions without accompanying images can yield comparable or even superior accuracy ^(31), (32)^. The true multimodal capabilities of reasoning models that integrate image and text inputs remain unclear ^(33), (34)^. Our findings suggest that current systems may still face important challenges in interpreting complex nephrology cases and in integrating visual and textual information for clinical reasoning.

Despite overall high accuracy, some questions were missed by all models, often involving Japan-specific guidelines or epidemiological data. These findings likely reflect insufficient alignment between LLMs and locally adapted guidance that depends on Japan-specific clinical information. This underscores a key limitation of LLMs trained primarily on English-language or broadly international data ^(35), (36)^. Consistent with these findings, a systematic review of 45 studies across 17 countries reported that GPT-4 achieved high overall accuracy but performed significantly better on examinations from English-speaking countries and that translating questions into English improved ChatGPT-3.5 but had no effect on GPT-4, underscoring persistent language- and context-dependent variability in performance ^(37)^. Improving reliability will require mechanisms that reference local guidelines, cite sources, and indicate uncertainty. Retrieval-augmented generation could help address this, but quality control and verification are essential to ensure clinical safety ^(38), (39)^.

While clinical deployment faces important challenges, these findings may also support a role for reasoning models as supplementary educational tools. For example, as one possible feature, some reasoning models can provide structured explanations or intermediate reasoning steps, which may help learners evaluate not only the correct answer but also the underlying reasoning ^(40)^. However, the structure and reliability of these explanations and reasoning steps may vary across models, and because this study did not formally assess their educational suitability, their effectiveness for this purpose remains uncertain.

Several limitations were identified in the design and evaluation. First, the evaluation was limited to MCQs, constraining generalizability to open-ended, multiturn clinical tasks ^(4)^. MCQ-based designs can sometimes be solved through pattern recognition rather than genuine clinical reasoning. Prior reports using alternative assessment formats suggest that current LLMs may struggle when information is incomplete, conflicting, or clinically subtle, underscoring the need to evaluate not only whether they choose the right answer but also how they interpret and weigh clinical clues ^(41), (42)^. Additionally, although question annotations used in the subgroup analyses were finalized by consensus between two authors, formal interrater agreement statistics were not calculated, which represents a potential source of uncertainty in the category-based interaction analyses. Furthermore, this study compared reasoning models against comparator models within each vendor family rather than directly toggling reasoning on or off within the same model; accordingly, the observed performance differences may also reflect factors beyond reasoning-related behavior, including model generation; release timing; and differences in scale, architecture, and training. This study thus represents a cross-sectional comparison between particular model versions rather than a direct test of reasoning capability. Higher MCQ accuracy should not be interpreted as direct evidence of clinical effectiveness; even in educational contexts, incorrect or misleading explanations may require expert supervision. Second, the SAQ-NBR dataset is publicly available, so data leakage into training corpora cannot be excluded ^(43)^. In addition, the evaluation was restricted to Japanese nephrology questions and within-family comparisons, which limits external validity. Furthermore, complete raw API request logs were not retained; therefore, some default parameter values applied to the exact model snapshots used could not be directly verified, and the values summarized in Supplementary Table S1 are based on publicly available vendor documentation. Third, GPT-5 was evaluated later than GPT-4o and the Gemini models, and the observed differences may partly reflect vendor-side updates or changes in served model behavior over time rather than stable model-class differences. Future studies using contemporaneous within-model contrasts, such as different reasoning modes or reasoning-effort settings, would allow for fairer and more reproducible comparisons when isolating the contribution of reasoning-related behavior. Fourth, we performed a single-run evaluation for each model and did not assess variability across repeated runs. Prior studies have suggested that repeated-run assessments can provide additional insight into response stability and reproducibility ^(44), (45), (46)^. Additionally, the qualitative error analysis was exploratory and based on author interpretation; therefore, it may be subject to some degree of subjectivity.

In conclusion, reasoning models outperformed comparator models on nephrology board–style MCQs, with advantages that varied by question type. These findings support further evaluation of reasoning models as supplementary tools for nephrology board–recertification preparation, while noting that these comparisons were cross-sectional and that direct clinical use requires task-specific validation.

## Article Information

### Acknowledgments

We thank the Japanese Society of Nephrology for making the Self-Assessment Questions for Nephrology Board Renewal (SAQ-NBR) question sets publicly available online and colleagues who provided feedback on the study design and analysis. Portions of the manuscript (translation and language editing) were assisted by an artificial intelligence tool. All authors reviewed and take full responsibility for the content.

### Author Contributions

Fumiya Kitano: conceptualization, data curation, formal analysis, and writing – original draft; Mamoru Masaki, Daisuke Ichikawa, and Yugo Shibagaki: writing – review and editing; and Ryunosuke Noda: project supervision, conceptualization, data curation, formal analysis, and writing – review and editing.

### Conflicts of Interest

None

### Data Availability and Transparency

The SAQ-NBR materials are available through the official Japanese Society of Nephrology website but are copyrighted works of the Society. Therefore, we are not permitted to redistribute the raw question set or official answer set through this study. The analysis code supporting the findings of this study is available from the corresponding author upon reasonable request.

## Supplement

Supplementary Material
