# Supplementary material for: Performance of Reasoning and Comparator Large Language Models on Nephrology Multiple-choice Questions
Source: JMA J. 2026 Aug 14;9(5):1322–31. doi: 10.31662/jmaj.2025-0603 (PMC13639661; doi:10.31662/jmaj.2025-0603)
Supplement: Supplementary file 1 [file 2433-3298-9-5-1322-s001.pdf]

Supplementary Table S1. Default Parameters Applied When Only the Model Identifier Was Specified in the API Call.

**Models**	**Exact model**	**Temperature**	**Top-p**	**Top-k**	**Reasoning effort**	**Verbosity**	**Candidate count**
GPT-4o	gpt-4o-2024-11-20	1.0	1.0	N/A	N/A	N/A	N/A
GPT-5	gpt-5-2025-08-07	Unknown	Unknown	N/A	Medium	Medium	N/A
Gemini 2.0 Flash	gemini-2.0-flash-001	1.0	0.95	64	N/A	N/A	1
Gemini 2.5 Pro	gemini-2.5-pro-preview-03-25	Unknown	Unknown	Unknown	Unknown	N/A	Unknown

Notes: The parameters were not explicitly set in the study API calls; only the model identifier was specified. The values shown therefore reflect vendor-documented defaults applied when parameters are omitted, where publicly documented.

API: application programming interface; N/A: the parameter was not applicable to that model family or was not part of the documented API configuration; Unknown: no explicit official documentation was identified for the exact model/version used.
